# Prospective study to assess the tissue response to HPC-coated p48 flow diverter stents compared to uncoated devices in the rabbit carotid artery model

**DOI:** 10.1186/s41747-019-0128-z

**Published:** 2019-12-05

**Authors:** Tim Lenz-Habijan, Pervinder Bhogal, Catrin Bannewitz, Ralf Hannes, Hermann Monstadt, Andreas Simgen, Ruben Mühl-Benninghaus, Wolfgang Reith, Hans Henkes

**Affiliations:** 1 phenox GmbH, Bochum, Germany; 2 Department of Interventional Neuroradiology, The Royal London Hospital, Whitechapel Road, London, E1 1BB UK; 3 Department of Neuroradiology, Saarland University, Homburg, Saar Germany; 4 Neuroradiological Clinic, Klinikum Stuttgart, Stuttgart, Germany; 5 Medical Faculty, University Duisburg-Essen, Essen, Germany

**Keywords:** Carotid artery (common), Foreign-body reaction, Flow diverter, Intracranial aneurysm, Polymers, Stents

## Abstract

**Background:**

Flow diverters (FDs) are widely used in the treatment of intracranial aneurysms, but the required medication increases the risk of haemorrhagic complications and limits their use in the acute setting. Surface modified FDs may limit the need for dual antiplatelet therapy (DAPT). Hydrophilic polymer coating (HPC) may reduce the need of medication.

**Methods:**

This explorative study, approved by the local authorities and the local welfare committee, compared stent behaviour and overall tissue response between HPC-coated FDs and uncoated FDs, both implanted into the common carotid arteries of eight New Zealand white rabbits. Endothelialisation, inflammatory response, and performance during implantation were assessed. Angiographic follow-up was performed to observe the patency of the devices after implantation and after 30 days. Histological examinations were performed at 30 days to assess foreign body reaction and endothelialisation. Kruskal-Wallis and Wilcoxon tests were used to compare non-parametric variables.

**Results:**

Angiography showed that both coated and uncoated FDs performed well during implantation. All devices remained patent during immediate follow-up and after 30 days. Histopathology showed no significant difference in inflammation within the vessel wall between the two cohorts (2.12 ± 0.75 *vs.* 1.96 ± 0.79, *p* = 0.7072). Complete endothelialisation of the stent struts was seen with very similar (0.04 ± 0.02 mm *vs.* 0.04 ± 0.03 mm, *p* = 0.892) neoendothelial thickness between the two cohorts after 30 days.

**Conclusion:**

Taking into account the limitation in sample size, non-significant differences between the HPC-coated and uncoated FDs regarding implantation, foreign body response, and endothelialisation were found.

## Key points

- A hydrophilic coating can be applied to flow diverters.
- The hydrophilic polymer coating (HPC) coating does not elicit an acute inflammatory response.
- The HPC coating does not alter the endothelialisation process.

## Background

The introduction of flow diverter (FD) stents allowed the treatment of aneurysms that were previously difficult to treat or untreatable. Aneurysms are excluded through a gradual process of intra-aneurysmal thrombosis and endothelialisation along the strands of the device [1]. In 2013, the *Pipeline for Uncoilable or Failed Aneurysms* trial [2] showed a 6-month occlusion rate of 73.6% (78/106) with progressive aneurysm occlusion, reaching 95% (60/63) at 5-year follow-up [3]. Although this trial specifically used the pipeline embolisation device (PED; Medtronic, Irvine, CA, USA), similar results have been obtained using other devices [4, 5].

One of the principle concerns when using endovascular stents and FDs is the need for dual antiplatelet therapy (DAPT). This is necessary in order to prevent thromboembolic complications. However, there is naturally an increased haemorrhagic risk. Ajadi et al. [6] recently performed a meta-analysis on studies published between 2013 and 2018 to determine the predictive value of the preoperative platelet reactivity units (PRU). They identified 12 studies that met the study criteria including data from 1464 PED cases and demonstrated that preprocedural hypo-response, defined as a PRU ranging from > 200 to > 240, carried an increased risk of thrombotic events with an absolute risk of 15%. Conversely, a hyper-response, defined as PRU < 60 to < 70, conferred an increased risk of haemorrhage with an absolute risk of 12%. In addition to the problems of predictability in response to antiplatelet medications, drug interactions can also interfere with the activity of these medications [7].

Similar to drug eluting stents that have been used in the cardiology and peripheral endovascular space for many years, surface coated stents have recently been developed for the neurovascular arena. These surface coatings are being designed to limit the necessity for DAPT and the thrombotic potential of the implants. The first of these devices was the PED Shield (Medtronic, Irvine, CA, USA), a device with a 3-nm-thick covalently bound phosphorylcholine surface modification. Phosphorylcholine is a major component of the outer membrane of erythrocytes and has demonstrated efficacy in resisting platelet adhesion as well as intimal hyperplasia [8–10]. The phenox hydrophylic polymer coating (pHPC) is a proprietary coating technology developed by phenox GmbH (Bochum, Germany), which also shows antithrombogenic properties when applied nitinol surfaces. The purpose of this explorative study was to assess the effects of implantation of the p48 movable wire (MW) HPC in the rabbit carotid artery model compared with a similar uncoated device, the p48 MW, with regard to implantation, overall tissue response, inflammation, healing and neointima formation.

## Methods

The study was approved by the local authorities and the local welfare committee and was performed under the licence number 07/14. Sample sizes where chosen according to fit the requirements of EN ISO10993-6 (Tests for local effects after implantation) (https://www.iso.org/standard/61089.html) in order to fit the requirements of our notified body. Overall a comparative study with uncoated and coated devices was performed in 8 New Zealand white rabbits for 30 days. The study period was chosen to assess foreign body reaction and ingrowth at an early stage.

### Animal experiments and premedication

Eight New Zealand white rabbits, of similar age (20 ± 4 weeks, mean ± standard deviation) and weight (4.125 ± 0.45 kg, mean ± standard deviation; range 3.4–4.8 kg), were given acetylsalicylic acid (10 mg/kg/day) and clopidogrel (10 mg/kg/day), via their drinking water. All animals received the DAPT starting 3 days prior to the procedures.

The supra-aortic vessels of the rabbit were of a suitable calibre for the deployment of the p48 FD as well as having a similar calibre to typical intracranial vessels treated with flow diversion in humans. In addition, this animal model has been extensively used in the assessment of neurovascular devices previously and is a widely accepted model [11]. Previous studies have tested HPC in different animal species [12].

### Stent implant procedure

All procedures were performed with the animals under general anaesthesia with Ketamin (60 mg/kg)/Rompun 2% (6 mg/kg) IM and maintenance with Ketamin (60 mg/kg)/Rompun 2% (6 mg/kg) in 10 mL NaCl at a flowrate of 2.5 mL/h via the ear vein. After surgical exposure of the right common femoral artery a 4Fr introducer sheath was inserted. Digital subtraction angiography of the common carotid arteries (CCAs) was performed with a 4Fr vertebral catheter (Glidecath, Terumo Europe, Leuven, Belgium). All the procedures were conducted under fluoroscopy and DSA by means of a single-arm angiographic system in posterior-anterior projection at two frames per second (Ziehm Vision imaging, Nuremberg, Germany). Prior to selective catheterisation and stent implantation the animals were fully heparinised to achieve an activated clotting time (2–2.5 times normal). A combination of 0.014 in. pORTAL microwire (Phenox GmbH, Bochum, Germany) and 0.021-in. Trevo Pro 18 microcatheter (Stryker, Kalamazoo, USA) or Prowler Select plus (Codman Neurovascular, West Chester, USA) was used to access the CCAs where deployment of the p48 MW and p48 MW HPC devices was performed. Acetylsalicylic acid and clopidogrel were continuously provided for the time until final angiography and sacrifice and are applied by the drinking water.

### Stent characteristics

The p48 MW FD is constructed from 48 braided drawn filled tubes (DFT) with each DFT strand constructed from a platinum filled nitinol tube. For p48 MW HPC, each DFT strand is coated with pHPC. The delivery system has a central, independently moveable wire and is compatible with 0.021-in. inner diameter (ID) microcatheters. The movable inner wire is made of nitinol with an atraumatic distal tip to prevent rupture. The p48 MW and p48 MW HPC is available in different sizes and is designed to treat vessels between 1.75 and 3 mm.

### HPC surface coating

The pHPC surface coating is a hydrophilic surface modification with antithrombogenic properties. Recently, two stent devices with this coating received the CE mark (pCONUS HPC and p48 MW HPC, phenox GmbH, Bochum, Germany). The coating is less than 12-nm thin, dense (as determined by x-ray photoelectron spectroscopy analysis) and covalently bound to the surface.

The thin nature of the coating does not affect the mechanical properties of the DFT strands or the device as a whole.

*In vitro* testing showed a significant reduction in the adherence of immunofluorescent CD61+ platelets on pHPC coated electropolished nitinol test plates and braided devices when incubated with whole blood from healthy volunteers compared with uncoated ones. Scanning electron microscopy demonstrated minimal adherent platelets on the coated test plates and FDs whereas a thick layer of adherent platelets was seen on uncoated specimen [13].

Previous *in vivo* studies have shown that the HPC does not affect the neoendothelialisation of the pCONUS neck bridging device in rabbits nor does it elicit either an acute or chronic inflammatory reaction within the vessel wall nor intima hyperplasia was observed when assessed at 30 and 180 days, respectively [14].

### Implant location and stent sizing

In each animal the uncoated p48 MW was implanted into the left CCA and the coated p48 MW HPC was implanted into the right CCA. The size of the implanted device was based upon the maximum diameter of the CCA at the site of the planned implantation on either side. The size of each device was calculated based on the two-dimensional angiographic imaging. The same size p48 MW HPC and p48 MW was implanted in each animal. In total, four p48 MW HPC 300-15, four p48 MW 300-15, four p48 MW HPC 200-12, and four p48 MW 200-12 were implanted.

### Angiographic follow-up

Control angiography was performed following implantation of the devices and 30 days after implantation. Selective angiography at final follow-up, using a 4Fr vertebral catheter, was performed via arteriotomy of the left common femoral artery.

### Harvest and gross imaging

Euthanasia by pentobarbital (Narcoren, Merial GmbH, Hallbergmoos, Germany) overdose (6–8 mL) was performed at 30 days whilst the animals were under anaesthesia with Ketamin (60 mg/kg)/Rompun 2% (6 mg/kg) in 10 mL NaCl at a flow rate of 2.5 mL/h via the ear vein. Each of the arterial segments in which either a p48 MW or p48 MW HPC were implanted were surgically resected and then fixed in 10% formaldehyde following which they were photographed and radiographed using a LX-60 cabinet radiography system (Faxitron, AZ, USA).

### Histological preparation and morphological assessment

After gross imaging, the excised arterial segments were dehydrated in a graded series of ethanol following which they were embedded in Spurr’s epoxy resin. After polymerisation, transverse sections from the proximal, middle, and distal third of each device shaft were removed and the cross sections adhered to plastic slides. The slides were prepared to a thickness of < 100 μ (Exakt, Oklahoma City, USA). After polishing of the slides they were stained with haematoxylin and eosin.

### Histiological assessment

An independent, experienced observer (CV Path, Gaithersburg, MD, USA) used digital planimetry with a calibrated camera to perform the morphometric analysis. The morphometric analysis included an assessment of the luminal area of the vessel, the area of the internal elastic laminae (IEL) and external elastic lamina (EEL) and the neointimal thickness which was measured as the distance from the IEL to luminal border. Semiquantitative data including medial fibrin and calcification deposition, inflammatory cell invasion into the media and adventitia, and the thickness of the adventitia were recorded (Table 1). The inflammation score was based on both the extent and the significance of inflammatory cell infiltration within each layer of the vessel wall (adventitia, media, and neointima). Final inflammatory scores were determined using the scale in Table 2. The giant cell reaction was recorded using the same scale.

**Table 1:** Semiquantitative histology scores

Attribute	Score	Description of assigned score
Injury score	0	No injury
	1	< 25% of the vessel circumference showing disruption of the EEL, IEL, or media respectively
	2	25–50% of the vessel circumference showing disruption of the EEL, IEL, or media respectively
	3	> 50% of the vessel circumference showing disruption of the EEL, IEL, or media respectively
Thrombus (platelets/fibrin)	0	None
	1	Minimal focal
	2	Mild multifocal
	3	Moderate regionally diffuse
	4	Severe marked diffuse total occlusion
Neointimal/medial fibrin deposition	0	None to focal interstitial fibrin minimal spotting of fibrin generally consistent with background levels
	1	< 10% of circumference showing interstitial fibrin
	2	10–25% of circumference showing interstitial fibrin
	3	> 25% of the circumference showing interstitial fibrin
Red blood cell extravasation score	0	No red blood cell extravasation
	1	< 25% red blood cell extravasation
	2	25–50% red blood cell extravasation showing disruption of the EEL, IEL, and media, respectively
	3	> 50% red blood cell extravasation
Calcification	0	None
	1	Focal with < 10% of the region affected
	2	Multifocal with 10–25% of the region affected
	3	Regionally diffuse with 26–30% of the region affected
	4	Regionally diffuse with > 30% of the region affected
Endothelial loss	0	None
	1	< 25% of the circumference
	2	25–50% of the circumference
	3	51–75% of the circumference
	4	> 75% of the circumference
Adventitial inflammation score	0	No inflammation to minimal interspersed inflammatory cells
	1	Mild peripheral inflammatory infiltration or focally moderated in < 25% of adventitial area
	2	Moderate peripheral inflammatory infiltration or focally marked in 25–50% of adventitial area
	3	Heavy peripheral inflammatory infiltration or focally marked in > 50% of adventitial area

Vessel wall injury was defined as a break in the arterial wall structures: external elastic lamina (EEL); internal elastic lamina (IEL); media. Neointimal fibrin deposition was defined as interstitial fibrin with or without associated stent mesh

**Table 2:** Inflammation severity score

Severity	Circumferential extension			
	< 25%	> 25–50%	> 50–70%	> 75%
0	0	0	0	0
1	1	1	1	1
2	1	1	2	2
3	2	2	3	4
4	2	3	4	4

Severity was defined as: 0 = no inflammation; 1 = rare inflammatory cells present; 2 = mild infiltrate, not being the predominant component of the associated tissue; 3 = moderate infiltrate; 4 = severe circumferential infiltrate (circumferential or near circumferential)

An independent pathologist (CV Path, Gaithersburg, MD, USA), blinded to the coating status of the p48 MW, performed the histopathological analysis. All slides and prepared stent sections were examined by light microscopy (× 20 as the maximum magnification). The presence of giant cells and granulomas on the stent struts was assessed and calculated as a percentage.

The media area (EEL area minus IEL area), neointimal area (IEL minus luminal are) and percent luminal stenosis (1 − [luminal area/IEL area] × 100) were also calculated.

### Statistics

Normal or near-normal data distributions were expressed as mean ± standard deviation. Paired *t* test comparisons were used to calculate the significance of differences between continuous variables of all parametric data using JMP software (version 13.0, Cary, NC). Nonparametric score data, including injury, fibrin (Table 1), and neointimal and adventitial inflammation were compared using a Wilcoxon test (JMP software version 13.0). A *p* value lower than 0.05 was considered significant.

## Results

### Clinical findings initially after implantation and after 30 days

Implantation of all devices was successful; all implants were fully expanded, with no in-stent stenosis and no thrombus formation visible. There was no angiographic evidence of impaired blood flow. All animals survived until the end of the study (30 days). Angiography at 30-day follow-up showed both implants fully expanded with no in-stent stenosis, no visible thrombus formation and the unimpeded blood flow in all vessels.

**Fig. 1:**
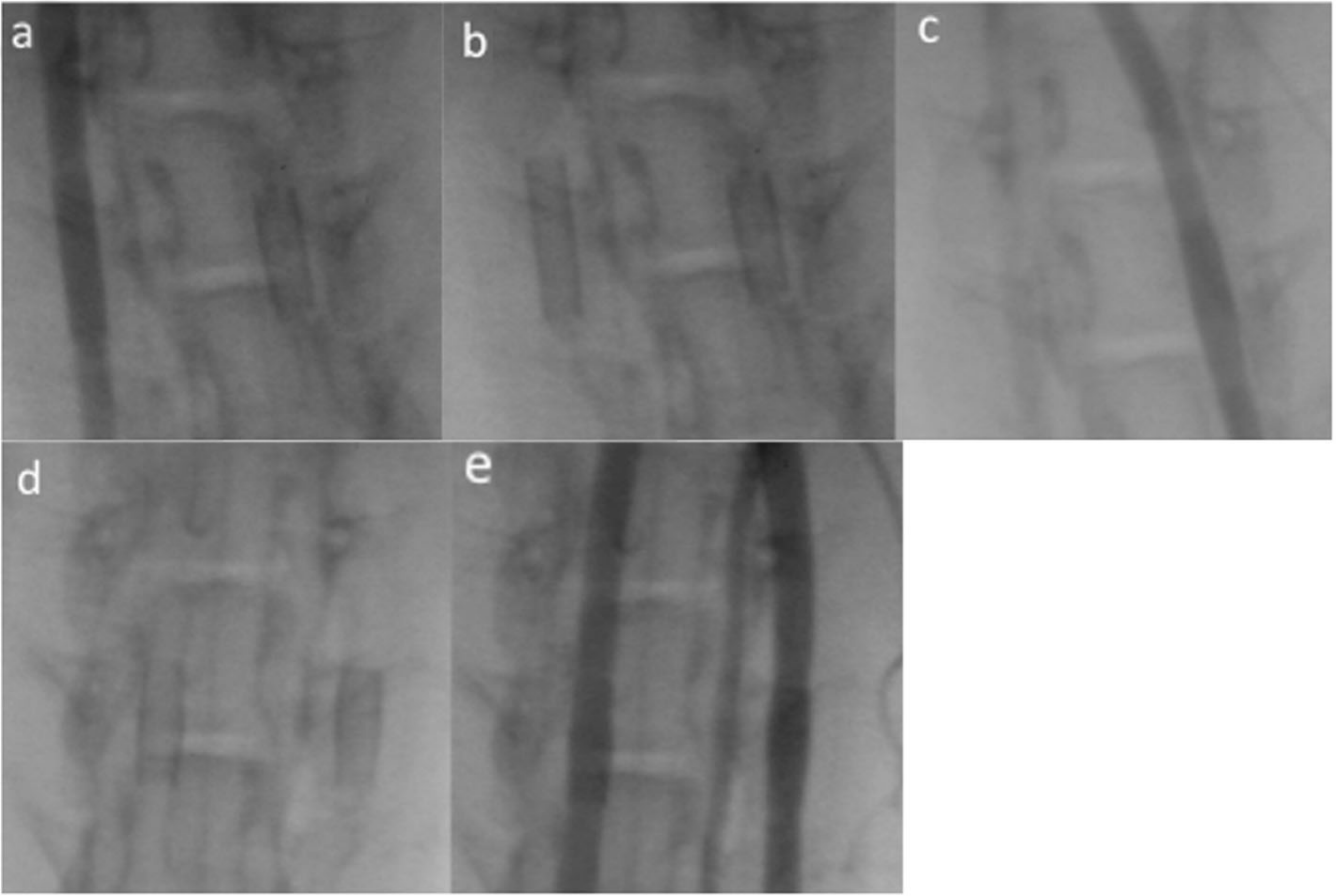
Exemplary angiographic results of animal 5, with p48 MW 200-12 implanted into the left (**a**) and p48 MW HPC-200-12 into the right carotid common artery (**c**), initially after implantation (**a**, **b** and **c**) and at 30-day follow-up (**d** and **e**). Both stents remain well opposed to the vessel wall with no evidence of stent migration or constriction (**d**). No in-stent stenosis, no thrombus formation and no obstruction of the blood flow is visible (**d** and **e**)

**Fig. 2:**
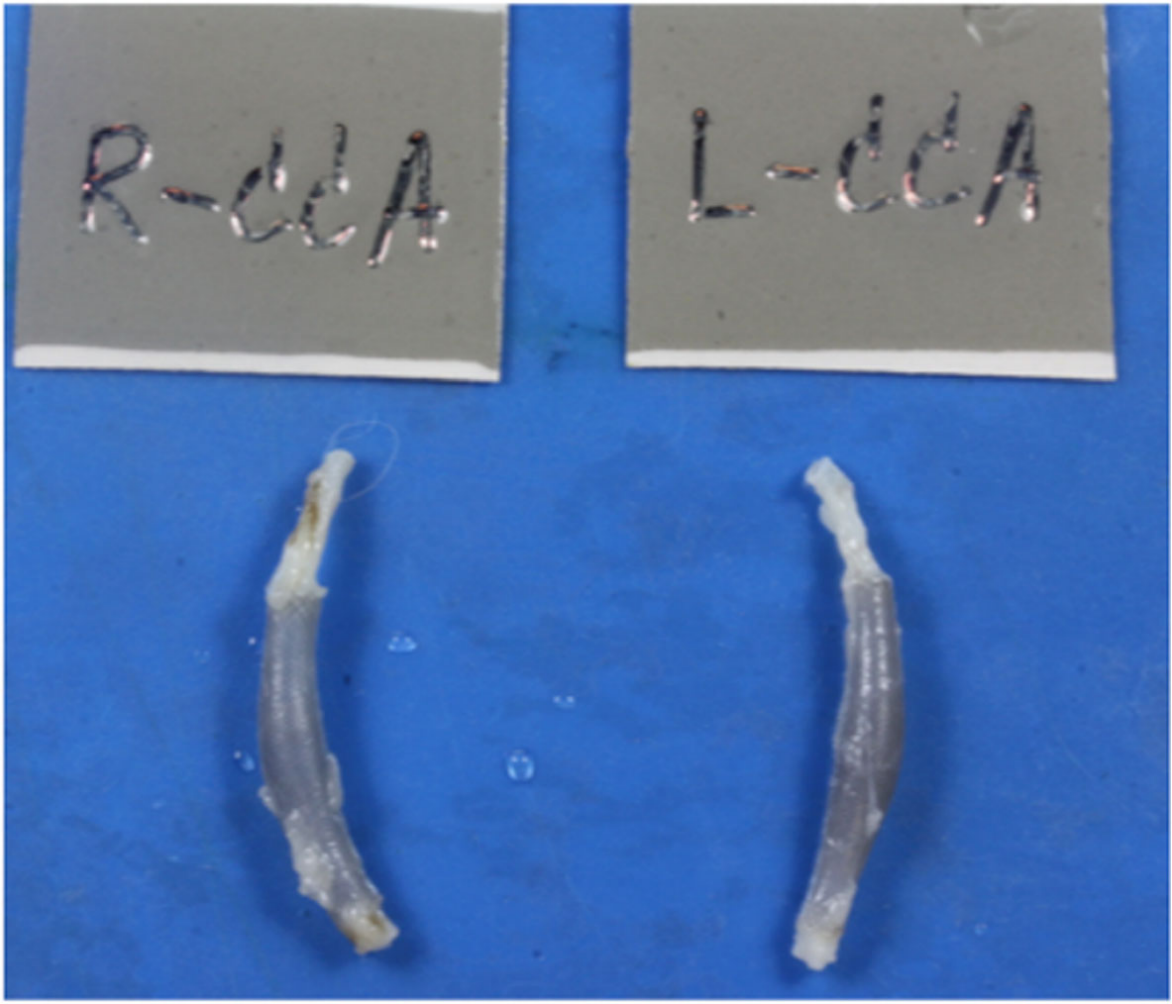
On gross evaluation (animal 1), there was no evidence of abnormality with either the flow diverter stents or the resected vessels

**Fig. 3:**
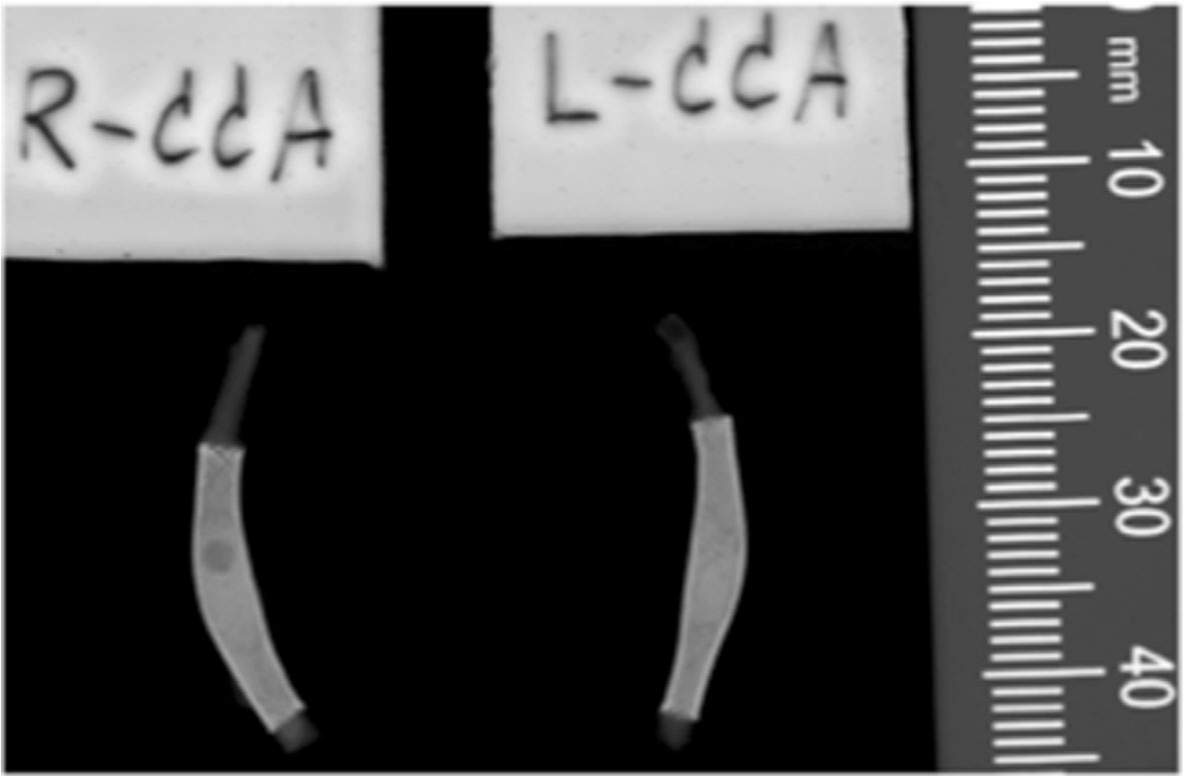
Faxitron images of the flow diverter stents in the left (p48 MW) and right (p48 MW HPC) rabbit common carotid arteries of animal 1. Both devices are evenly expanded with no evidence of mesh fractures. The caudal end of the common carotid artery and stent is at the top of the image

Figure 1 shows the angiographic results of one animal, with p48 MW 200-12 in the left CCA and p48 MW HPC 200-12 in the right CCA, initially after implantation and at 30-day follow-up. In total, 16 stents and associated CCAs were available for morphological and histological analysis. On gross evaluation, there was no evidence of obvious abnormality (Fig. 2). Examples of Faxitron imaging of implanted p48 MW and p48 MW HPC are shown in Fig. 3.

**Fig. 4:**
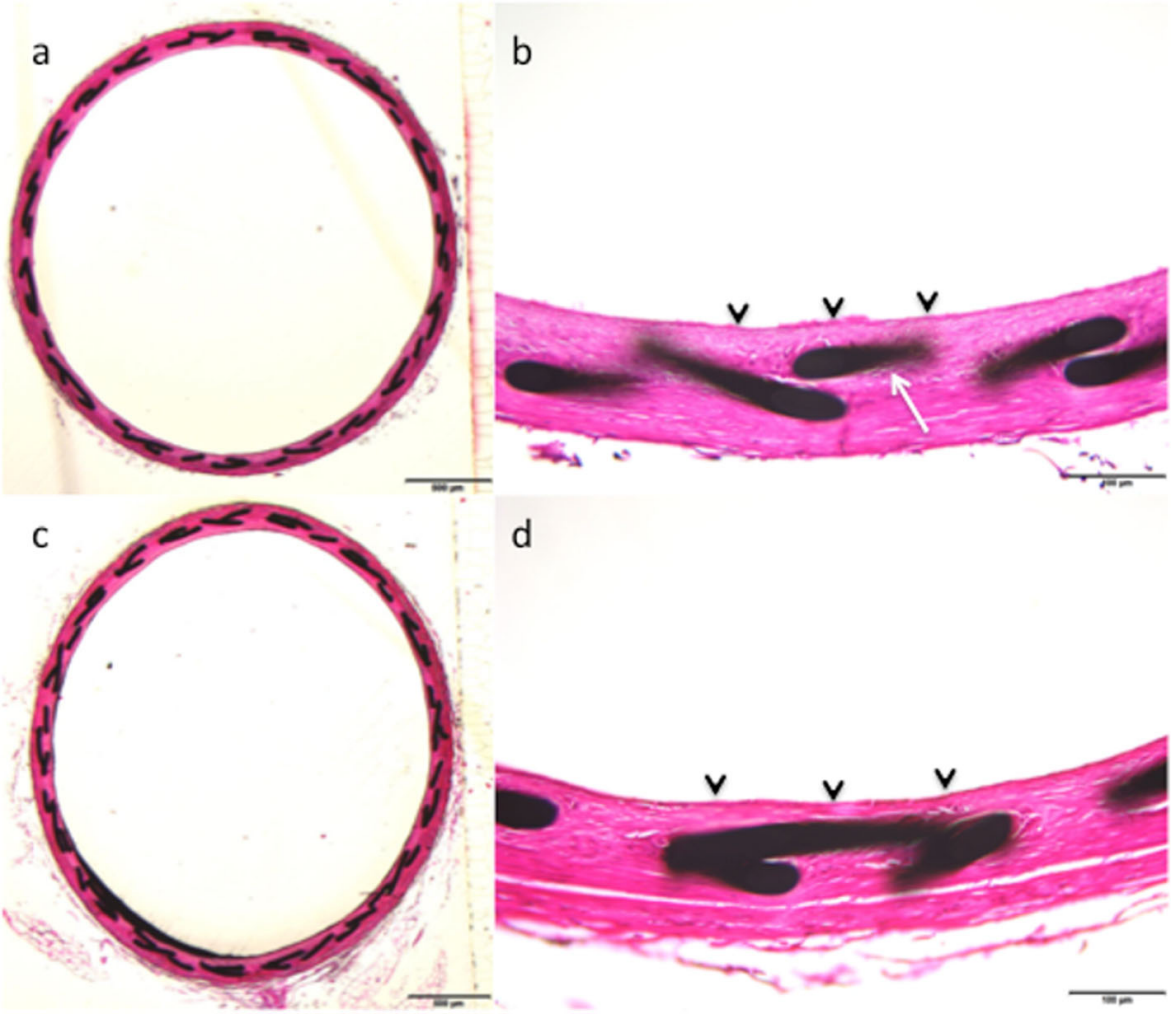
Histological samples from animal 4 show good apposition of the p48 MW HPC with the arterial wall (**a**) with the braided wires of the device (**b**, white arrow) covered by a thin neointimal layer (**b**, black arrowheads). The p48 MW has a similar appearance within the vessel (**c**) as well as at higher power magnification (**d**) with a thin neointimal layer seen (**d**, black arrowheads)

### Morphological and histological analysis

The morphometric findings between the two groups were similar. There was no statistically significant difference between the luminal area of the vessel, IEL, or EEL area, neointimal area or neointimal thickness. Importantly, there was no evidence of a statistically significant difference in the degree of stenosis between the p48 MW and p48 MW HPC (Fig. 4). The results are summarised in Table 3.

**Table 3:** Morphometric comparison of cross-sectional vessel areas and neointimal thickness

Implant group	EEL area (mm^2^)	IEL area (mm^2^)	Lumen area (mm^2^)	Medial area (mm^2^)	Neointimal area (mm^2^)	Stenosis (%)	Neointimal thickness (mm)
p48 MW HPC (*n* = 8)	5.97 ± 0.64	5.55 ± 0.58	4.78 ± 0.51	0.42 ± 0.08	0.77 ± 0.19	13.76 ± 2.79	0.04 ± 0.02
Uncoated P48 MW (*n* = 8)	5.73 ± 0.86	5.30 ± 0.84	4.55 ± 0.79	0.43 ± 0.06	0.75 ± 0.24	14.31 ± 4.30	0.04 ± 0.03
*p* value	0.526	0.501	0.499	0.936	0.865	0.766	0.892

Data are presented as mean ± standard deviation. *EEL* External elastic lamina, *IEL* Internal elastic lamina

There was no evidence of greater inflammation within the intima, media or adventitia between the two groups. There was no evidence of a statistically different inflammation score between the two groups or of a greater number of giant cells. Neointimal coverage appeared to be complete in both cohorts. The results are summarised in Table 4.

**Table 4:** Histologic comparison of vessel injury and healing

Implant group	Injury score	Thrombus score	Intimal/medial fibrin score	Inflammation score	Giant cells score	RBCs score	Intimal coverage	Adventitial inflammation score
p48 MW HPC (*n* = 8)	0.46 ± 0.43	0.04 ± 0.12	0.00 ± 0.00	2.12 ± 0.75	2.04 ± 0.84	0.00 ± 0.00	Complete	0.13 ± 0.25
Uncoated P48 MW (*n* = 8)	0.54 ± 0.35	0.00 ± 0.00	0.00 ± 0.00	1.96 ± 0.79	1.96 ± 0.79	0.00 ± 0.00	Complete	0.08 ± 0.24
*p* value	0.737	0.317	1.000	0.707	0.789	1.000	NA	0.589

Data are presented as mean ± standard deviation. *RBC* Red blood cell, *NA* Not available

At histological analysis, all devices were well expanded without evidence of luminal thrombosis, although some sections showed postmortem clots, likely from incomplete washout of blood. All devices were fully incorporated by a smooth-muscle cell rich neointima and stenosis values were minimal and similar between test (p48 MW HPC, 13.76 ± 2.79, mean ± standard deviation) and control (uncoated p48 MW = 14.31 ± 4.30, *p* = 0.766). There was no/minimal fibrin and no evidence of haemorrhage or calcification. The majority of devices, irrespective of the coating showed circumferential to near circumferential mild to moderate chronic inflammation nearest the mesh, including giant cells (inflammation scores: p48 MW HPC, 2.12 ± 0.75; uncoated p48 MW, 14.31 ± 4.30, *p* = 0.707). Endothelial coverage over luminal surfaces was mostly complete (Fig. 3).

## Discussion

The purpose of this explorative study was to demonstrate the similarity of the p48 MW HPC FD compared with the uncoated p48 MW FD with regard to overall tissue response, inflammation, healing and neointima formation.

Following the injury to the arterial wall after stent deployment, there is a triphasic cellular response [15–17]. This initially involves platelet activation and an inflammatory response, followed by granulation tissue and the migration and proliferation of smooth cells and finally tissue remodelling. This process on endothelialisation can take up to 6 weeks. Various different processes are involved in the eventual endothelialisation and repair of the vessel wall with much of the information on the tissue response derived from studies involving coronary arteries [18–21].

It is known from previous studies assessing pHPC, that this coating technology has strong antithrombogenic properties on braided wire constructs, such as FD stents, as well as laser cut stents *in vitro* [12–14].

First of all, the described antithrombogenic effect of pHPC could not be observed *in vivo* in this study, due to the lack of a negative control. The administered DAPT did efficiently prevent thrombus formation on the uncoated p48 MW. However, Colgan et al. [22] published the first in man case of a p48 MW HPC. They reported the use of two telescoped p48 MW HPCs to treat a patient with a subarachnoid haemorrhage secondary to a vertebral artery dissection. In this case single antiplatelet therapy (SAPT) was used and there was no evidence of stent thrombosis or stroke. The patient made a complete recovery. Further clinical studies are currently on going.

It is known from other surface modified FDs on the market, that the development of the neointima can be influenced by the device surface quality. In a recent study by Matsuda et al. [23], the neointima formation on the surface of the PED Shield was compared with that on the surface of the PED Flex and Solitaire (Medtronic). In this *in vivo* study, the devices were implanted into the carotid arteries of pigs and the development of neointima was assessed using optical coherence tomography. These authors showed that there was a trend towards earlier endothelialisation on the PED Shield and that by day 21 the neointimal ratio ([mean stent area minus mean lumen area]/mean stent area) was significantly higher for both the PED Flex and PED Shield than for the Solitaire (*p* < 0.05 and *p* < 0.01, respectively). Similarly, the neointimal thickness ratio (minimal neointimal thickness/maximum neointimal thickness) was significantly greater for PED Shield than for PED Flex and Solitaire (*p* < 0.05 and *p* < 0.01, respectively). These results suggest that the PED Shield is associated with greater neointimal formation and more concentric neointimal formation compared with the other devices tested and hence that surface modifications to FDs and stents may alter the tissue response.

Given the nature of the pHPC and its ability to inhibit thrombocyte adhesion, it is not inconceivable that this same property may inhibit endothelial cell adhesion and proliferation. Therefore, one big question of the current study was whether pHPC influences the endothelialisation and neointima formation on coated devices compared with uncoated ones. Our results suggest that, even after only 30 days, both devices were equally well covered with neointima. This indicates the noninterference of pHPC with endothelial cell proliferation. These *in vivo* results demonstrate that neither p48 MW nor p48 MW HPC elicit an acute inflammatory response or an acute fibrotic reaction. Also there was no evidence to suggest neoendothelialisation of the p48 MW HPC occurred at a different rate to the uncoated p48 MW.

Several limitations and the translation into clinical practice need to be considered carefully. The follow-up period (30 days) was too short to observe acute body reactions and the early endothelialisation, but a previous study [14] did not show a chronic inflammatory response within the arterial wall on delayed histological assessment at 180 days. Similarly, FDs are designed to treat aneurysms and so the interaction of the p48 MW HPC with developing intra-aneurysmal thrombus is unknown. Furthermore, as coils are sometimes used as adjunctive devices when coiling is performed the interaction between coils, particularly coils that are not bare platinum, is unknown. Larger cohorts with longer follow-up periods are required. Furthermore, studies assessing the thrombogenicity of these devices under SAPT use should also be performed.

In conclusion, we showed through an *in vivo* study on animal model that the p48 MW HPC FD stent does not elicit an acute inflammatory response greater or significantly different from that of the uncoated p48 MW FD stent.

## Data Availability

There is no further data available to share at this time.

## Abbreviations

CCA: Common carotid artery
DAPT: Dual antiplatelet therapy
DFT: Drawn filled tubes
EEL: External elastic lamina
FD: Flow diverter
IEL: Internal elastic lamina
MW: Movable wire
PED: Pipeline embolisation device
pHPC: Phenox hydrophylic polymer coating
PRU: Platelet reactivity units
SAPT: Single antiplatelet therapy

## References

[1] Kadirvel Ramanathan, Ding Yong-Hong, Dai Daying, Rezek Issa, Lewis Debra A., Kallmes David F. (2014). Cellular Mechanisms of Aneurysm Occlusion after Treatment with a Flow Diverter. Radiology.

[2] Becske T, Kallmes DF, Saatci I (2013). Pipeline for uncoilable or failed aneurysms: results from a multicenter clinical trial. Radiology.

[3] Becske T, Brinjikji W, Potts MB (2017). Long-term clinical and angiographic outcomes following pipeline embolization device treatment of complex internal carotid artery aneurysms: five-year results of the pipeline for uncoilable or failed aneurysms trial. Neurosurgery.

[4] Wakhloo A. K., Lylyk P., de Vries J., Taschner C., Lundquist J., Biondi A., Hartmann M., Szikora I., Pierot L., Sakai N., Imamura H., Sourour N., Rennie I., Skalej M., Beuing O., Bonafe A., Mery F., Turjman F., Brouwer P., Boccardi E., Valvassori L., Derakhshani S., Litzenberg M. W., Gounis M. J. (2014). Surpass Flow Diverter in the Treatment of Intracranial Aneurysms: A Prospective Multicenter Study. American Journal of Neuroradiology.

[5] Pierot L, Spelle L, Berge J (2019). SAFE study (safety and efficacy analysis of FRED embolic device in aneurysm treatment): 1-year clinical and anatomical results. J Neurointerv Surg.

[6] Ajadi Ebunoluwa, Kabir Shaowli, Cook Aaron, Grupke Stephen, Alhajeri Abdulnasser, Fraser Justin F (2019). Predictive value of platelet reactivity unit (PRU) value for thrombotic and hemorrhagic events during flow diversion procedures: a meta-analysis. Journal of NeuroInterventional Surgery.

[7] Martinez-Moreno R., Aguilar M., Wendl C., Bäzner H., Ganslandt O., Henkes H. (2015). Fatal Thrombosis of a Flow Diverter due to Ibuprofen-related Antagonization of Acetylsalicylic Acid. Clinical Neuroradiology.

[8] Chen C, Lumsden AB, Ofenloch JC (1997). Phosphorylcholine coating of ePTFE grafts reduces neointimal hyperplasia in canine model. Ann Vasc Surg.

[9] Chen Changyi, Ofenloch John C., Yianni Yiannakis P., Hanson Stephen R., Lumsden Alan B. (1998). Phosphorylcholine Coating of ePTFE Reduces Platelet Deposition and Neointimal Hyperplasia in Arteriovenous Grafts. Journal of Surgical Research.

[10] Campbell EJ, O’Byrne V, Stratford PW (1994). Biocompatible surfaces using methacryloylphosphorylcholine laurylmethacrylate copolymer. ASAIO J.

[11] Fahed Robert, Raymond Jean, Ducroux Célina, Gentric Jean-Christophe, Salazkin Igor, Ziegler Daniela, Gevry Guylaine, Darsaut Tim E. (2016). Testing flow diversion in animal models: a systematic review. Neuroradiology.

[12] Martínez Moreno R, Bhogal P, Lenz-Habijan T (2019). In vivo canine study of three different coatings applied to p64 flow-diverter stents: initial biocompatibility study. Eur Radiol Exp.

[13] Lenz-Habijan Tim, Bhogal P., Peters Marcus, Bufe Albrecht, Martinez Moreno Rosa, Bannewitz Catrin, Monstadt Hermann, Henkes Hans (2018). Hydrophilic Stent Coating Inhibits Platelet Adhesion on Stent Surfaces: Initial Results In Vitro. CardioVascular and Interventional Radiology.

[14] Bhogal Pervinder, Lenz-Habijan Tim, Bannewitz Catrin, Hannes Ralf, Monstadt Hermann, Simgen Andreas, Mühl-Benninghaus Ruben, Reith Wolfgang, Henkes Hans (2019). The pCONUS HPC: 30-Day and 180-Day In Vivo Biocompatibility Results. CardioVascular and Interventional Radiology.

[15] Mitra AK, Agrawal DK (2006). In stent restenosis: bane of the stent era. J Clin Pathol.

[16] Grewe Peter H, Deneke Thomas, Machraoui Abderrahman, Barmeyer Jürgen, Müller Klaus-Michael (2000). Acute and chronic tissue response to coronary stent implantation: pathologic findings in human specimen. Journal of the American College of Cardiology.

[17] Inoue Teruo, Croce Kevin, Morooka Toshifumi, Sakuma Masashi, Node Koichi, Simon Daniel I. (2011). Vascular Inflammation and Repair. JACC: Cardiovascular Interventions.

[18] Welt Frederick GP, Tso Colin, Edelman Elazer R., Kjelsberg Michael A, Paolini John F, Seifert Philip, Rogers Campbell (2003). Leukocyte recruitment and expression of chemokines following different forms of vascular injury. Vascular Medicine.

[19] Otsuka Fumiyuki, Finn Aloke V., Yazdani Saami K., Nakano Masataka, Kolodgie Frank D., Virmani Renu (2012). The importance of the endothelium in atherothrombosis and coronary stenting. Nature Reviews Cardiology.

[20] Chaabane C, Otsuka F, Virmani R, Bochaton-Piallat ML (2013). Biological responses in stented arteries. Cardiovasc Res.

[21] Campbell JH, Campbell GR (2012). Smooth muscle phenotypic modulation--a personal experience. Arterioscler Thromb Vasc Biol.

[22] Colgan Frances, Aguilar Pérez Marta, Hellstern Victoria, Reinhard Matthias, Krämer Stefan, Bäzner Hansjörg, Ganslandt Oliver, Henkes Hans (2018). Vertebral Artery Aneurysm: Ruptured Dissecting Aneurysm, Implantation of Telescoping p48_HPC Flow Diverter Stents under Antiaggregation with ASA Only. The Aneurysm Casebook.

[23] Matsuda Yoshikazu, Chung Joonho, Lopes Demetrius K (2017). Analysis of neointima development in flow diverters using optical coherence tomography imaging. Journal of NeuroInterventional Surgery.

